# *CTNNBIP1-CLSTN1* functions as a housekeeping chimeric RNA and regulates cell proliferation through SERPINE2

**DOI:** 10.1038/s41420-023-01668-8

**Published:** 2023-10-07

**Authors:** Chen Chen, Fujun Qin, Sandeep Singh, Yue Tang, Hui Li

**Affiliations:** 1 School of Basic Medical Sciences, Academy of Medical Sciences, Zhengzhou University, Zhengzhou, 450001 Henan China; 2 Academy of Medical Sciences, Zhengzhou University, Zhengzhou, 450052, Henan, China; 3 Department of Pathology, School of Medicine, University of Virginia, Charlottesville, VA 22908 USA; 4 ICMR-Center for Research, Management and Control of Haemoglobinopathies (Unit of ICMR-National Institute of Immunohaematology, Mumbai), Chandrapur, Maharashtra 442406 India; 5 Present Address: Department of Clinical Laboratory, Qilu Hospital of Shandong University Dezhou Hospital, Dezhou, 253000 Shandong China

**Keywords:** Gene expression, RNA splicing, Transcriptomics

## Abstract

The conventional understanding that chimeric RNAs are unique to carcinoma and are the products of chromosomal rearrangement is being challenged. However, experimental evidence supporting the function of chimeric RNAs in normal physiology is scarce. We decided to focus on one particular chimeric RNA, *CTNNBIP1-CLSTN1*. We examined its expression in various tissues and cell types and compared it quantitatively among cancer and noncancer cells. We further investigated its role in a panel of noncancer cells and investigated the functional mechanism. We found that this fusion transcript is expressed in almost all tissues and a wide range of cell types, including fibroblasts, epithelial cells, stem cells, vascular endothelial cells, and hepatocytes. In addition, the *CTNNBIP1-CLSTN1* expression level in noncancerous cell lines was not evidently different from that in cancer cell lines. Furthermore, in at least three cell types, silencing *CTNNBIP1-CLSTN1* significantly reduced the cell proliferation rate by inducing G2/M arrest and apoptosis. Importantly, rescue experiments confirmed that cell cycle arrest was restored by exogenous expression of the chimera but not the wild-type parental gene. Further evidence is provided that *CTNNBIP1-CLSTN1* regulates cell proliferation through *SERPINE2*. Thus*, CTNNBIP1-CLSTN1* is an example of a new class of fusion RNAs, dubbed “housekeeping chimeric RNAs”.

## Background

The discovery of the *BCR-ABL* fusion gene by Nowell et al. [1] during the 1960s, found to be a result of chromosomal translocation in chronic myelogenous leukemia, provided a strategy for the search for gene fusions involved in various neoplasms [2, 3]. Even since then, the prevailing view is that gene fusions are cancer specific and that they lead to the production of fusion products (RNAs and proteins), which can also be used as specific biomarkers and drug targets [4, 5]. This assumption has resulted in the rapid expansion of the Mitelman Database of Chromosome Aberrations and Gene Fusions in Cancer in the Cancer Genome Anatomy Project. However, this dogma has been challenged when an increasing number of studies have led to the continuous discovery of chimeric RNAs involved in normal physiology, especially stemming from the availability of a large number of transcriptome sequencing (RNA-seq) databases [6]. The prevalence of chimeric RNAs has thus far exceeded our expectations [7–9]; these RNAs have several characteristic modalities [7, 10], and at least some of them also play key roles in various settings [7, 8, 11, 12]. In particular, a group of chimeric RNAs has been suggested to play a housekeeping role, in that their expression is generally ubiquitous and are indispensable for cell survival and maintenance.

We previously implemented and analyzed approximately 300 RNA sequencing libraries established from 30 different nonneoplastic human tissues and cell lines, which led to the discovery of 9778 chimeric RNAs that are potential products of cis-splicing of adjacent genes (cis-SAGe) or RNA trans-splicing [7]. In that study, the vast majority of the chimeric RNAs were found to be tissue specific, and approximately 10% were detected in more than one sample, of which 51 were detected in more than five different samples. Among these recurrent chimeras, *CTNNBIP1-CLSTN1* was found in a large number of tissues and seemed to be indispensable for growth of an immortalized astrocyte line in culture [7]. In addition, we demonstrated that *CTNNBIP1-CLSTN1* is a cis-SAGe chimeric RNA [13] composed of two neighboring parental genes located on chr1p36 [7, 13] and is induced when the transcription factor CTCF is silenced [9]. However, the exact role the chimeric RNA plays and the mechanism of its functionality are not clear.

We hypothesized the existence of a group of chimeric RNAs functioning as “housekeeping” chimeric RNAs, i.e., playing an universal role in maintaining physiological functions in various noncancer cell types in humans. In this study, we aimed to investigate whether the *CTNNBIP1-CLSTN1* chimeric RNA is a member of this new class of chimeric RNAs and to further investigate its downstream pathway. We first confirmed the presence of this transcript in different types of tissues and cells, and from these, we then selected three different cell lines to study the roles of the chimera in relation to cell proliferation, migration and apoptosis. Transcriptome sequencing and downstream analyses were further conducted to uncover its functional mechanism.

## Results

### *CTNNBIP1-CLSTN1* is widely expressed across human tissues and cell types

**Fig. 1:**
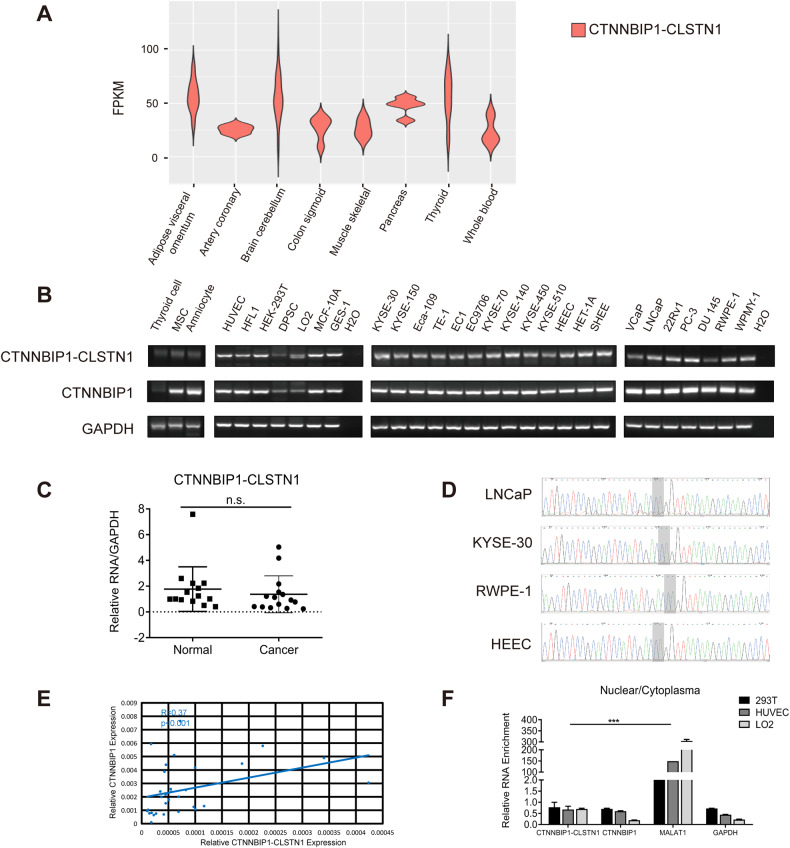
The expression of *CTNNBIP1-CLSTN1* in various human tissues and cell lines. **A** The expression of *CTNNBIP1-CLSTN1* in the whole Genotype-Tissue Expression (GTEx) database. FPKM values of the chimeric RNA in various tissues were plotted. **B** RT‒PCR analysis of the expression of *CTNNBIP1-CLSTN1* in several human cell lines, followed by gel electrophoresis. The internal control *GAPDH* and the wild-type parental gene *CTNNBIP1* were also included. **C** The expression level of the chimera was measured in 15 noncancer cell lines and 15 cancer cell lines using qRT‒PCR. The expression of the fusion was normalized to the internal control *GAPDH*. **D** Sanger sequencing of RT‒PCR products in the cancer cell lines LNCaP and KYSE-30 and the noncancer cell lines RWPE-1 and HEEC. The light gray area denotes the fusion junction site. **E** The correlation between the relative expression level of the fusion and *CTNNBIP1* was plotted (*R* = 0.37, *P* < 0.001). **F** HEK-293T cells, HUVECs and LO2 cells were subjected to nuclear and cytoplasmic fractionation. The expression levels of the chimera and *CTNNBIP1* in each fraction were measured by qRT‒PCR. *GAPDH* and the known long noncoding RNA *MALAT1* were used as controls. The ratios of expression in the nucleus and cytoplasm were plotted. The asterisks indicate statistical significance: *P < 0.05. **P < 0.01. ***P < 0.001.

Previously, we identified 291 recurrent chimeric RNAs by analyzing RNA-Seq data from approximately 300 noncancer tissues and cells [7]. *CTNNBIP1-CLSTN1* was one of the identified chimeric RNAs and was detected by RT‒PCR in the liver, lung, kidney, etc. We recently expanded our chimeric RNA search to the whole Genotype-Tissue Expression (GTEx) database, which contains 9495 nondiseased human tissue samples from 53 different tissues [14, 15]. From this study, we found that *CTNNBIP1-CLSTN1* was detected in almost all samples (Fig. 1A and Supplementary Fig. S1). Except for the a few tissues including brain, its expression is not significantly different among different tissues (Supplementary Table S1).

We then performed RT‒PCR to detect the fusion and its parental genes in 15 noncancer cell lines, including fibrocytes, amniocytes, epithelial cells, stem cells, and vascular endothelial cells, of 12 different tissues of origin, and separated the products by gel electrophoresis (Fig. 1B). *CTNNBIP1-CLSTN1* was detected in all the samples. Using qRT‒PCR, we quantified its expression in 15 noncancer cells and 15 esophageal and prostate cancer cell lines. There were no statistically significant differences in the expression level between the cancer cell and normal cell lines (Fig. 1C, and Supplementary Figs. S2 and S3). The junction sequence was shown to be exactly the same across different cancer cell lines (LNCaP and KYSE-30), and noncancer lines (RWPE-1 and HEEC) by Sanger sequencing (Fig. 1D). We also evaluated the wild-type parental transcripts using primers covering at least one exon not shared with the fusion transcript. Although the wild-type *CTNNBIP1* transcript was readily detectable, the wild-type *CLSTN1* transcript was not. We also found that the relative expression levels of the chimeric fusion and *CTNNBIP1* were positively correlated (Pearson’s R = 0.37, *P* < 0.001) (Fig. 1E, and Supplementary Figs. S2 and S3), consistent with the chimeric RNA being a product of 5’ gene read-through and suggesting that the vast majority of *CLSTN1* transcripts are used to form chimeric RNA.

This chimeric RNA is predicted to encode an in-frame chimeric protein. Thus, we decided to take advantage of the observation that regular protein-coding mRNAs localize mainly in the cytoplasm, while long noncoding RNAs often localize in the nucleus to regulate transcription [16–18]. We chose HEK-293T human embryonic kidney cells, human umbilical vein endothelial cells (HUVECs), and LO2 hepatocytes for fractionation and extracted RNA to detect the chimeric RNA by qRT‒PCR. The classical protein-coding gene *GAPDH* and long noncoding RNA *MALAT1* were used as controls. Not surprisingly, *MALAT1* was most abundant in the nuclear fraction. In contrast, *CTNNBIP1-CLSTN1*, wild-type *CTNNBIP1* and *GAPDH* were all enriched in the cytoplasmic fraction (Fig. 1F). This observation is consistent with a previous Western blot analysis [7], further supporting the hypothesis that the fusion has a protein-coding function.

### Silencing *CTNNBIP1-CLSTN1* reduced cell proliferation and cell motility

**Fig. 2:**
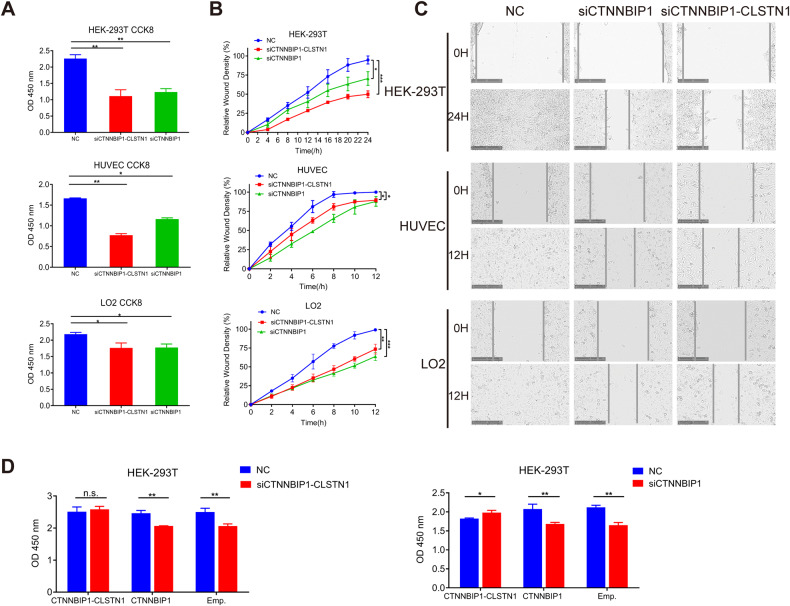
Knocking down *CTNNBIP1-CLSTN1* results in significant reductions in cell proliferation and cell motility in HEK-293T cells, HUVECs and LO2 cells. **A** A CCK8 assay was used to determine the cell proliferation rate 72 h after siRNA transfection. Both siRNAs resulted in significant suppression of proliferation in the three cell lines. **B** Both siRNAs resulted in a significant reduction in cell motility in the three cell lines. **C** Representative micrographs of cells transfected with the NC siRNA, siCTNNBIP1-CLSTN1 and siCTNNBIP1 showed migration across a wound at zero (upper panel) and 12 h (lower panel) for HUVECs and LO2 cells and at zero (upper panel) and 24 h (lower panel) for HEK-293T cells. The scale bars represent 250 μm. **D** In a rescue experiment, HEK-293T cells were transfected with plasmids expressing *CTNNBIP1-CLSTN1*, plasmids expressing *CTNNBIP1* or the empty control plasmid, and 24 h later, the cells were transfected with siCTNNBIP1-CLSTN1, siCTNNBIP1 or the negative control (NC) siRNA. Cell viability was measured by a CCK8 assay after another 48 h. The asterisks indicate statistical significance: *P < 0.05; **P < 0.01; ***P < 0.001.

Based on its ubiquitous expression pattern, we hypothesized that the chimeric RNA may belong to the group of chimeric RNAs that we collectively call “housekeeping chimeric RNAs”. Indeed, a previous report documented its indispensable role in immortalized astrocytes [7]. To further investigate the function of *CTNNBIP1-CLSTN1* and support its basic role in principal cell maintenance, we transfected siRNAs targeting the chimera into multiple human noncancer cell lines, namely, HEK-293T, HUVEC and LO2. The siRNA siCTNNBIP1-CLSTN1, targeting the fusion junction sequence, resulted in a significant reduction in the level of the fusion transcript but not the wild-type *CTNNBIP1* transcript in all three cell lines (Supplementary Figs. S4 and S5). Since we could not design a siRNA that specifically silences wild-type *CTNNBIP1*, as a control, we designed a siRNA (siCTNNBIP1) that targets a sequence common to the fusion and wild-type *CTNNBIP1*. As predicted, this siRNA led to silencing of both the fusion and wild-type *CTNNBIP1* (Supplementary Fig. S5). Both siCTNNBIP1-CLSTN1 and siCTNNBIP1 significantly decreased cell proliferation, based on the CCK8 assay (Fig. 2A). Cell migration was monitored by a wound healing assay with live-cell imaging microscopy after wounding. The relative wound closure percentage was calculated by measuring the density of cells that migrated into the original wound. Both siCTNNBIP1-CLSTN1 and siCTNNBIP1 significantly inhibited cell migration compared to the negative control siRNA (Fig. 2B). Figure 2C shows representative micrographs of HUVEC and LO2 cell migration across a wound at zero and 12 h and of HEK-293 cell migration at zero and 24 h. These results suggest that *CTNNBIP1-CLSTN1* plays an important role in general cell growth and motility, independent of cell type.

To rule out a potential off-target effect of the siRNAs, we performed rescue experiments by transfecting the *CTNNBIP1-CLSTN1*-expressing plasmid and the construct encoding wild-type *CTNNBIP1*. The reduced cell proliferation was indeed reversed by the *CTNNBIP1-CLSTN1* construct in both the siCTNNBIP1-CLSTN1 and siCTNNBIP1 groups. In contrast, wild-type *CTNNBIP1* failed to reverse the reduction in cell proliferation in either group (Fig. 2D), suggesting that the reduced cell proliferation was caused by silencing of the fusion but not of wild-type *CTNNBIP1*.

### Silencing *CTNNBIP1-CLSTN1* resulted in cell cycle arrest and apoptosis

**Fig. 3:**
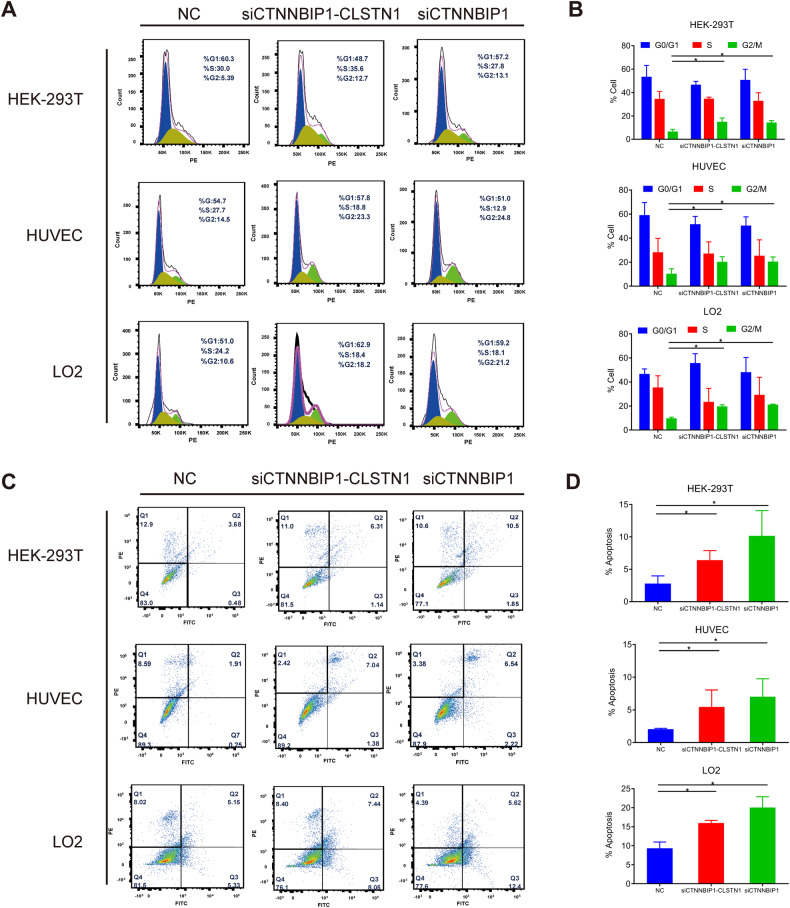
Knocking down *CTNNBIP1-CLSTN1* resulted in G2/M arrest and apoptosis. **A** The cell cycle distribution 72 h after siRNA transfection was examined by flow cytometry and analyzed by FlowJo. Representative pictures are shown. **B** Silencing *CTNNBIP1-CLSTN1* induced G2/M arrest. **C** Apoptosis was examined 72 h after siRNA transfection by flow cytometry and analyzed by FlowJo. Representative pictures are shown. **D** Silencing *CTNNBIP1-CLSTN1* induced apoptosis. The data are presented as the means ± SDs. The asterisks indicate statistical significance: *P < 0.05.

To investigate the mechanism underlying the reduced cell proliferation caused by silencing of the fusion, we performed flow cytometry with propidium iodide staining to evaluate the cell cycle after siRNA transfection. The numbers of cells in G2/M phase were notably increased in both siRNA groups with silencing of the fusion compared to those in the NC group (Fig. 3A). This G2/M arrest was observed in all three cell lines (Fig. 3B).

We then evaluated apoptosis using Annexin V-FITC and propidium iodide staining. The results from the flow cytometry assay showed that transfection of either siCTNNBIP1 or siCTNNBIP1-CLSTN1 noticeably increased apoptosis in all three cell lines compared to that in the corresponding control groups (Fig. 3C, D).

### Overexpression of *CTNNBIP1-CLSTN1* but not wild-type *CTNNBIP1* promotes cell growth and cell migration

**Fig. 4:**
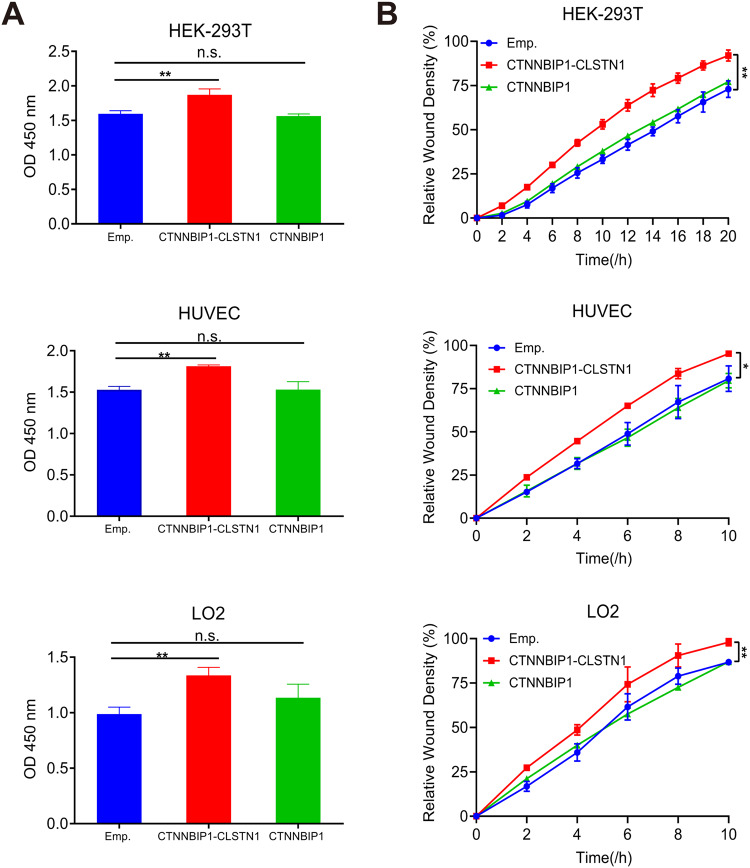
Overexpression of *CTNNBIP1-CLSTN1* but not wild-type *CTNNBIP1* promotes cell proliferation and cell motility. **A** HEK-293T cells, HUVECs and LO2 cells were transfected with pCDNA3.1 plasmids containing the full-length coding sequence of *CTNNBIP1-CLSTN1* (CTNNBIP1-CLSTN1), pCDNA3.1 plasmids containing the full-length coding sequence of *CTNNBIP1* (CTNNBIP1) or the corresponding empty vector (Emp.). A CCK8 assay was used to determine the cell proliferation rate 72 h after plasmid transfection. Overexpression of the fusion protein increased the cell proliferation rate, whereas overexpression of wild-type CTNNBIP1 had no such effect. **B** Overexpression of the fusion also promoted cell motility, whereas overexpression of wild-type *CTNNBIP1* had no such effect.

In this experiment, HEK-293T cells, HUVECs, and LO2 cells were transfected with either the pCDNA3.1 plasmid that encodes the full-length CDS of *CTNNBIP1-CLSTN1*, the pCDNA3.1 plasmid that encodes *CTNNBIP1*, or the corresponding empty vector. In contrast to the loss-of-function experiments, the CCK8 assay showed that the expression of the chimera but not wild-type *CTNNBIP1* promoted proliferation in all three cell lines (Fig. 4A). Consistently, the wound healing assay also demonstrated that the overexpression of *CTNNBIP1-CLSTN1* but not *CTNNBIP1* increased the migration ability in all three cell lines (Fig. 4B).

### *CTNNBIP1-CLSTN1* influences cell proliferation by regulating the autocrine signaling factor *SERPINE2*

**Fig. 5:**
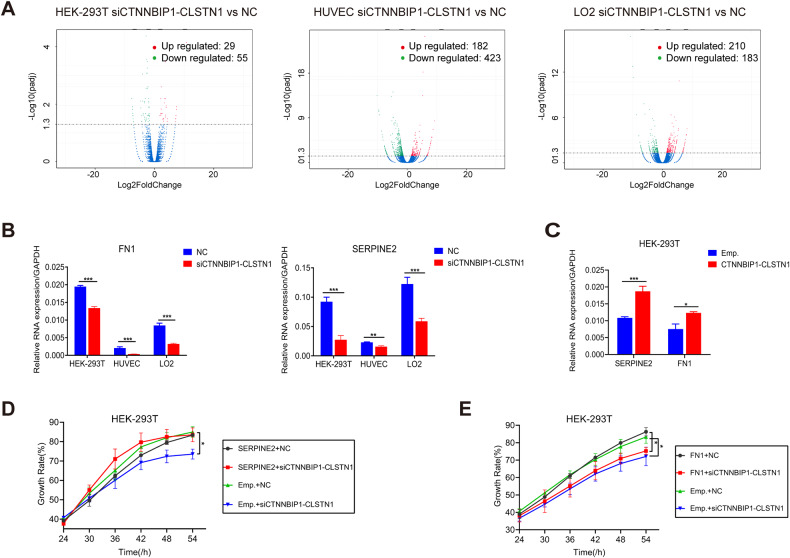
*CTNNBIP1-CLSTN1* regulates cell proliferation through *SERPINE2*. **A** Volcano plots of DEGs in HEK-293T cells, HUVECs, and LO2 cells. The red dots indicate significantly upregulated genes. The green dots indicate significantly downregulated genes. The blue dots indicate genes with no significant difference in expression. **B** qRT‒PCR analysis of the expression of *FN1* and *SERPINE2* 48 h after siRNA transfection. Silencing *CTNNBIP1-CLSTN1* significantly suppressed *FN1* and *SERPINE2*. **C** Overexpression of *CTNNBIP1-CLSTN1* increased the expression of *FN1* and *SERPINE2*. **D** In a rescue experiment, the reduction in the cell growth rate caused by siCTNNBIP1-CLSTN1 transfection was reversed by transfecting the cells with the *SERPINE2* expression plasmid. **E** Overexpression of *FN1* had no such rescue effect. The horizontal axis shows the time after plasmid transfection. The asterisks indicate statistical significance: *P < 0.05. **P < 0.01. ***P < 0.001.

To investigate the downstream pathway mediating the effect of *CTNNBIP1-CLSTN1* on cell proliferation, we performed transcriptome sequencing analysis of HEK-293T cells, HUVECs and LO2 cells transfected with siCTNNBIP1-CLSTN1 and the negative control siRNA. Differential expression analysis between the two groups was conducted in HEK-293T cells, HUVECs and LO2 cells, identifying 84, 605, and 393 differentially expressed genes (DEGs), respectively (Supplementary Table 2). A volcano plot of the DEGs in the three cell lines is shown in Fig. 5A (*p*adj < 0.05). Gene Ontology (GO) terms were also examined for enrichment of the DEGs. A total of 135 enriched GO terms in HEK-293T cells, 286 enriched GO terms in HUVECs, and 137 enriched GO terms in LO2 cells were found (Supplementary Table 2). Several terms were consistent across these cell lines and were consistent with the general role the fusion plays. However, there were also specific GO terms unique to the individual cell lines, suggesting that the fusion may have additional roles that are cell type specific. The top 13 GO terms in HEK-293T cells, the top 23 in HUVECs, and the top 14 in LO2 cells are displayed (Supplementary Fig. S6).

Among the DEGs, fibronectin 1 (*FN1*) and serine protease inhibitor E2 (*SERPINE2*) were notable because they were both downregulated in the siCTNNBIP1-CLSTN1 group in all three cell lines. Furthermore, they were among the several GO terms related to cell growth (GO:0001558, GO:0040008, and GO:0016049). Fibronectin 1 (*FN1*) [19, 20] is a member of the glycoprotein family that is widely expressed in multiple cell types (GTEx data, Supplementary Fig. S7A). Serine protease inhibitor E member 2 (*SERPINE2*) [21, 22], also called Protease Nexin-1 (*PN-1*), belongs to the Serpin gene superfamily and is ubiquitously expressed in human tissues and cells (GTEx, Supplementary Fig. S7B). We first confirmed *FN1* and *SERPINE2* expression by qRT‒PCR (Fig. 5B). The expression levels of both were reduced upon silencing of the fusion. In contrast, overexpression of *CTNNBIP1-CLSTN1* upregulated both genes (Fig. 5C). We then investigated whether the effect of the fusion is mediated by these two genes. To this end, we transfected expression plasmids encoding *FN1* or *SERPINE2* into cells previously transfected with siCTNNBIP1-CLSTN1 or siCT. As indicated in Fig. 5D, *SERPINE2* restored the cell proliferation rate to a normal value. In contrast, *FN1* failed to rescue the reduction in cell growth induced by siCTNNBIP1-CLSTN1 (Fig. 5E), suggesting that the effect of the chimeric RNA on cell proliferation is mediated mostly through SERPINE2.

## Discussion

In recent years, the exploration of gene fusions and chimeric RNAs in various carcinomas has progressed considerably [23]. Numerous examples have been found by high-throughput approaches, including microarray and next-generation sequencing analyses—for instance, *TMPRSS2-ETS* [24, 25] and *D2HGDH-GAL3ST2* [26] in prostate cancer, *LHX6-NDUFA8* and *SLC2A11-MIF* in cervical cancer [27], *GOLM1-MAK10* in esophageal squamous cell carcinoma (ESCC) [28], *EML4-ALK* in non-small cell lung cancer (NSCLC) [29], *CHFR-GOLGA3* in bladder cancer [30], *RRM2-C2orf48* in colorectal cancer (CRC) [31], and ASTN2-PAPPA_as_ in esophageal cancer [32]. Most of these gene fusions are significantly overexpressed in cancer tissues and cells compared to the corresponding noncancer tissues and cells. In addition, some of them have been verified to have clinical correlations between their expression level and cancer stage and/or patient survival [26]. Without doubt, they represent effective markers for clinical diagnosis/prognosis and/or drug targets.

However, in recent years, continuous discoveries have demonstrated that chimeric RNAs are not a unique phenomenon in cancer, nor are all the products of gene fusion. Chimeric RNAs are widespread in normal human tissues and cells [7, 8, 33–36]. *JAZF1-JJAZ1* is observed in endometrial stromal cells, and instead of resulting from chromosome rearrangement, it is formed via RNA trans-splicing in these cells [37]. Similarly, *DUS4L-BCAP29*, which is a product of cis-splicing of adjacent genes, exists not only in prostate cancer and gastric cancer tissues, as previously reported [38, 39], but is also present in various normal tissues [40]. In a previous study, 291 fusion transcripts were found by analyzing nearly 300 RNA-Seq libraries, with *CTNNBIP1-CLSTN1* detected in five nonneoplastic tissues [7]. In another study, Singh et al. explored the landscape of chimeric RNAs in 9,495 Genotype-Tissue Expression (GTEx) samples and established a dataset containing a total of 7,193 chimeric RNAs [8], where *CTNNBIP1-CLSTN1* was detected in the majority of samples. The results we present here provide further evidence that *CTNNBIP1-CLSTN1* is commonly expressed in various cell types and is expressed at similar levels in normal cells and cancer cells. Furthermore, this fusion also plays basic physiological roles that sustain cell growth and cell migration. This evidence supports the classification of this fusion as a housekeeping chimeric RNA.

**Fig. 6:**
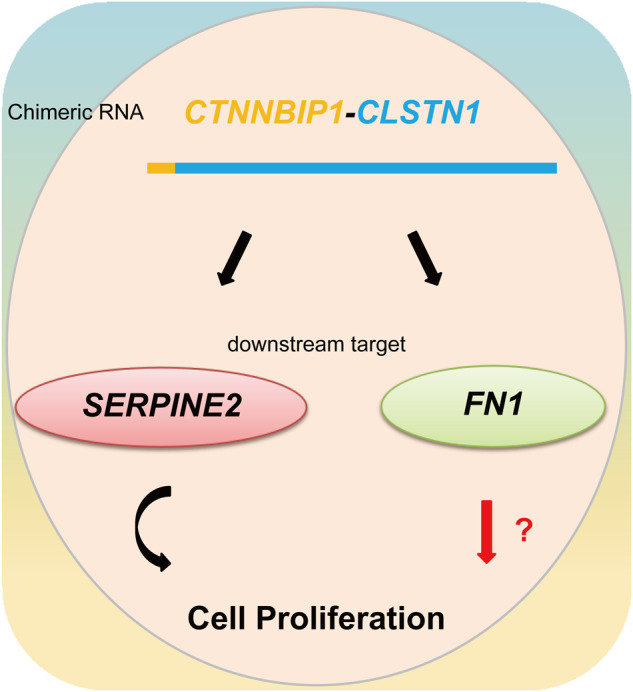
Schematic of the chimeric RNA *CTNNBIP1-CLSTN1 and its functional mechanism*. *CTNNBIP1-CLSTN1* regulates the expression of two downstream target genes, *SERPINE2* and *FN1*. Silencing the fusion reduces the cell proliferation rate, which can be restored by *SERPINE2* but not FN1. It is possible that *FN1* mediates other physiological roles of the fusion in cells.

Transcriptome sequencing analyses connected this fusion with downstream targets, including *FN1* and *SERPINE2*. *FN1* is an adhesive glycoprotein of the extracellular matrix with a variety of binding domains for cell surface and extracellular ligands [19, 20] and is involved in some biological processes, including cell viability and apoptosis, through these multiple interaction sites [41, 42]. *SERPINE2* is an extracellular plasminogen activator inhibitor that inhibits the activity of various proteases [21, 22] and functions in many physiological processes, including inflammation, cell growth and metastasis [43, 44]. They are both ubiquitously expressed in human tissues and cells (Fig. S7). Rescue experiments confirmed that the expression of *SERPINE2* can restore cell proliferation to a normal level, suggesting the role of *SERPINE2* in mediating the effect of *CTNNBIP1-CLSTN1* on cell proliferation. However, the expression of *FN1* failed to rescue cell proliferation, suggesting that *FN1* may play other roles (Fig. 6).

In addition to the common DEGs among the three different cell models, there were groups of differentially expressed genes that were found in only one or two cell models, suggesting that the fusion may also have cell-specific roles. The exact mechanism by which this fusion RNA regulates the expression of its downstream genes remains unclear and will be the direction of our future studies.

## Conclusion

In summary, *CTNNBIP1-CLSTN1* is ubiquitously expressed in normal and cancer cells. It regulates cell proliferation in various cell types and thus represents a new class of RNAs, housekeeping chimeric RNAs. We believe that more of these chimeric RNAs exist. Their presence challenges the traditional dogma that chimeric fusion RNAs are cancer specific and indicates the need for caution in expediting the clinical translation of fusion RNAs discovered in cancer tissue/cells into biomarkers and/or therapeutic targets.

## Methods

### Cell culture, siRNA and transfection

HEK-293T cells, HUVECs, and LO2 cells were purchased from American Type Culture Collection (ATCC, USA), and no further confirmation was conducted. They were maintained in DMEM/high-glucose medium with 4500 mg/L glucose and 4.0 mM L-glutamine (HyClone^TM^, USA) supplemented with 10% fetal bovine serum (LONSERA, Uruguay) and 1% penicillin and streptomycin (HyClone^TM^, USA). The cells were incubated at 37 °C in 5% CO_2_, and the medium was changed every other day. Cells were detached with 0.25% trypsin with 1 g/L EDTA (HyClone^TM^, USA). siRNAs were synthesized by Sangon Biotech (Shanghai, China) and transfected with Lipofectamine RNAiMAX (Life Technologies, USA) following the manufacturer’s protocols. Transfection efficiencies were evaluated 48 h after siRNA transfection.

The targeting sequences were as follows:

si-Negative Control, CGTACGCGGAATACTTCGA;

siCTNNBIP1, GGAAGAGTCCGGAGGAGAT;

siCTNNBIP1-CLSTN1, TGCTTGTTAACCTGGTCGA.

### RNA extraction, quantitative reverse transcription–PCR (qRT‒PCR) and Sanger sequencing

RNA was extracted from cells with a BEI-BEI BIOTECH Total RNA Isolation Kit (Zhengzhou, China) and reverse-transcribed by a Tiangen FastKing cDNA Kit (Beijing, China) according to the manufacturer’s instructions. The primers used to amplify the fusion have been described previously [13]. qRT‒PCR was carried out using the ABI StepOne Plus Real-Time PCR System (Life Technologies) with TB Green® Premix Ex Taq^TM^ (TaKaRa, Japan) prior to gel electrophoresis with Gel-Red^TM^ Nucleic Acid Gel Stain (Biotium, California, USA). Data were analyzed using the ΔΔCt method, and expression values were normalized to the internal control GAPDH. An Axygen® AxyPrep DNA Gel Extraction Kit (USA) was used for DNA purification, and validation by Sanger sequencing was then conducted at Sangon Biotech.

The primer sequences used for qRT‒PCR were as follows:

WT-CTNNBIP1: F, 5’-CTCATGCTGCGGAAGATGGGAT-3’; R, 5’-CTGGAAAACGCCATCACCACGT-3’;

FN1: F, 5’-CCACCCAATGTTCAGCTCAC-3’; R, 5’-GTAGCATCTGTCACACGAGC-3’;

SERPINE2: F, 5’-CTTCCTCTTGGCCTCTGTGA-3’; R, 5’-ACGCCGTATCTCATCACCAT-3’

### Construction of the *CTNNBIP1-CLSTN1* plasmid and plasmid transfection

The full-length cDNA of *CTNNBIP1-CLSTN1* and the wild-type *CTNNBIP1* open reading frame were cloned and inserted into the pCDNA3.1 plasmid and verified by Sanger sequencing. For transient expression, the plasmids were transfected into cells with Lipofectamine 2000 (Life Technologies, USA) following the manufacturer’s instructions.

### Cell proliferation assay

HEK-293T cells, HUVECs and LO2 cells were seeded in 96-well plates at the appropriate confluence. Seventy-two hours after siRNA or plasmid transfection, cell proliferation was measured with a DOJINDO Cell Counting Kit-8 (Japan), for which the cells were incubated at 37 °C in 5% CO_2_ for two hours. Cell viability in each well was determined by measuring the O.D. at 450 nm.

### Rescue experiment

For the CCK8 assay, HEK-293T cells were seeded in 96-well plates at 2,000 cells per well. The cells were first transfected with pCDNA3.1 plasmids expressing *CTNNBIP1-CLSTN1*, *CTNNBIP1*, *SERPINE2*, or *FN1* or empty control plasmid. All plasmids were synthesized by GenScript and confirmed by Sanger sequencing. Twenty-four hours later, the cells were transfected with siCTNNBIP1-CLSTN1, siCTNNBIP1 or the NC siRNA. Forty-eight hours after siRNA transfection, 10 µl CCK8 solution was added to each well and incubated for two hours at 37 °C in 5% CO_2_. Cell viability in each well was determined by measuring the O.D. at 450 nm. For live-cell imaging, HEK-293T cells were seeded and transfected as described for the cell proliferation assay. Then, the plates were placed under a live-cell imaging microscope (JULI Stage, NanoEnTek, Seoul, South Korea) after siRNA transfection for 60 h. Images were captured every six hours for measurement of cell proliferation. A total of 10 imaging cycles were performed. Image analysis was performed to analyze cell proliferation using JULI Stat (NanoEnTek, Seoul, South Korea), and a cell growth curve was plotted according to the cell density.

### Wound healing assay

HEK-293T cells, HUVECs and LO2 cells were seeded in 6-well plates and transfected with siRNAs or plasmids with the proper reagents. Approximately 48 h after transfection, when the cell confluence was more than 90%, a wound was created by manually scratching a longitudinal line along the bottom of each well with a 10 µl plastic pipette tip. The plates were rinsed twice with PBS, and the medium was replaced with fresh complete DMEM. After that, the plates were placed under a live-cell imaging microscope (JULI Stage, NanoEnTek, Seoul, South Korea) and incubated at 37 °C in 5% CO_2_. Images were captured every two hours, and the gap distance remaining after scratching was documented, with a total of 12 imaging cycles performed. Image analysis was performed for analysis fo wound healing using JULI Stat (NanoEnTek, Seoul, South Korea). A wound closure curve based on the percentage of cell migration measured as the repopulated wound area relative to the original wound area was plotted.

### Flow cytometry

Seventy-two hours after siRNA transfection, HEK-293T cells, HUVECs and LO2 cells were detached with 0.25% trypsin and washed twice with PBS, and the cell density was adjusted to 1 × 10^6^ cells/ml. Next, the cells were fixed with precooled 70% alcohol overnight at 4 °C, washed twice with PBS, and treated with 500 µl of a propidium iodide (PI) (KeyGEN BioTECH, Nanjing, China) reaction mixture containing RNase in the dark for 60 min at room temperature. Cell cycle phases were detected by flow cytometry (FACS Calibur, Becton, Dickinson and Company, New Jersey, USA), and the data were analyzed by FlowJo software. For apoptosis detection, cells were collected and washed as described above, mixed with 500 µl binding buffer containing 5 µl Annexin V-FITC and 5 µl PI staining solution (KeyGEN BioTECH, Nanjing, China), and incubated in the dark for 15 min at room temperature. A flow cytometer was used to detect apoptosis, and the data were analyzed by FlowJo.

### Cell fractionation assay

HEK-293T cells, HUVECs and LO2 cells were detached with 0.25% trypsin, washed once with PBS, and then separated into two fractions using NE-PER nuclear and cytoplasmic extraction reagents (Thermo Fisher, USA) following the manufacturer’s instructions. RNA from each fraction was extracted separately with a BEI-BEI BIOTECH Total RNA Isolation Kit, and qRT‒PCR was then performed with *MALAT1* and *GAPDH* as normalization controls.

### Statistical analysis

All data analysis was performed by SPSS 23.0 (IBM, Chicago, Illinois, USA). Data are shown as mean ± standard deviation (SD). The differences were analyzed using Student’s *t* test and one-way ANOVA. Pairwise comparison of *CTNNBIP1-CLSTN1* expresison between tissues was conducted by Mann–Whitney *U* test. *P* < 0.05 was considered statistically significant.

### Supplementary information

supplementary table 1

supplementary table 2

Supplementary figures

## Data Availability

The raw and processed RNA-sequencing data generated in this study have been uploaded to the NCBI Gene Expression Omnibus (GEO; http://www.ncbi.nlm.nih.gov/geo/) under accession number GSE165479. The security token for reviewers to access the data before it is publicly available is gjqlgkamtxgltgn. Flow cytometry data have been deposited into the Flow Repository. The ID and URL with the secret code for reviewer access are listed below: Cell cycle: FR-FCM-Z3E3 http://flowrepository.org/id/RvFrm5qG1S7akXGqSKb85uWhbRTZ0ACn5uCIPoGiulAygdG2Lq0SnQRYez7L5oHB Cell apoptosis: FR-FCM-Z3E4 http://flowrepository.org/id/RvFrzD1pnAaXP44GVUKW0wVl1QimdHNOKR1hXyc07ao0xIZODy0tzrEgOykrnMUgAll the other data supporting the findings of this study are available within the article and its supplementary information files or from the corresponding author upon reasonable request. A reporting summary for this article is available as a Supplementary Information file.
