# Supplementary material for: CTNNBIP1-CLSTN1 functions as a housekeeping chimeric RNA and regulates cell proliferation through SERPINE2
Source: Cell Death Discov. 2023 Oct 7;9:369. doi: 10.1038/s41420-023-01668-8 (PMC10560238; doi:10.1038/s41420-023-01668-8)
Supplement: Supplementary file 3 — Supplementary figures [file 41420_2023_1668_MOESM3_ESM.docx]

**Supplementary Figure S1. The expression of *CTNNBIP1-CLSTN1* in the whole Genotype-Tissue Expression (GTEx) database.** The chimeric RNA *CTNNBIP1-CLSTN1* was detected in almost all samples. The violin plot shows the FPKM values of the fusion transcript in different tissues/cell types.


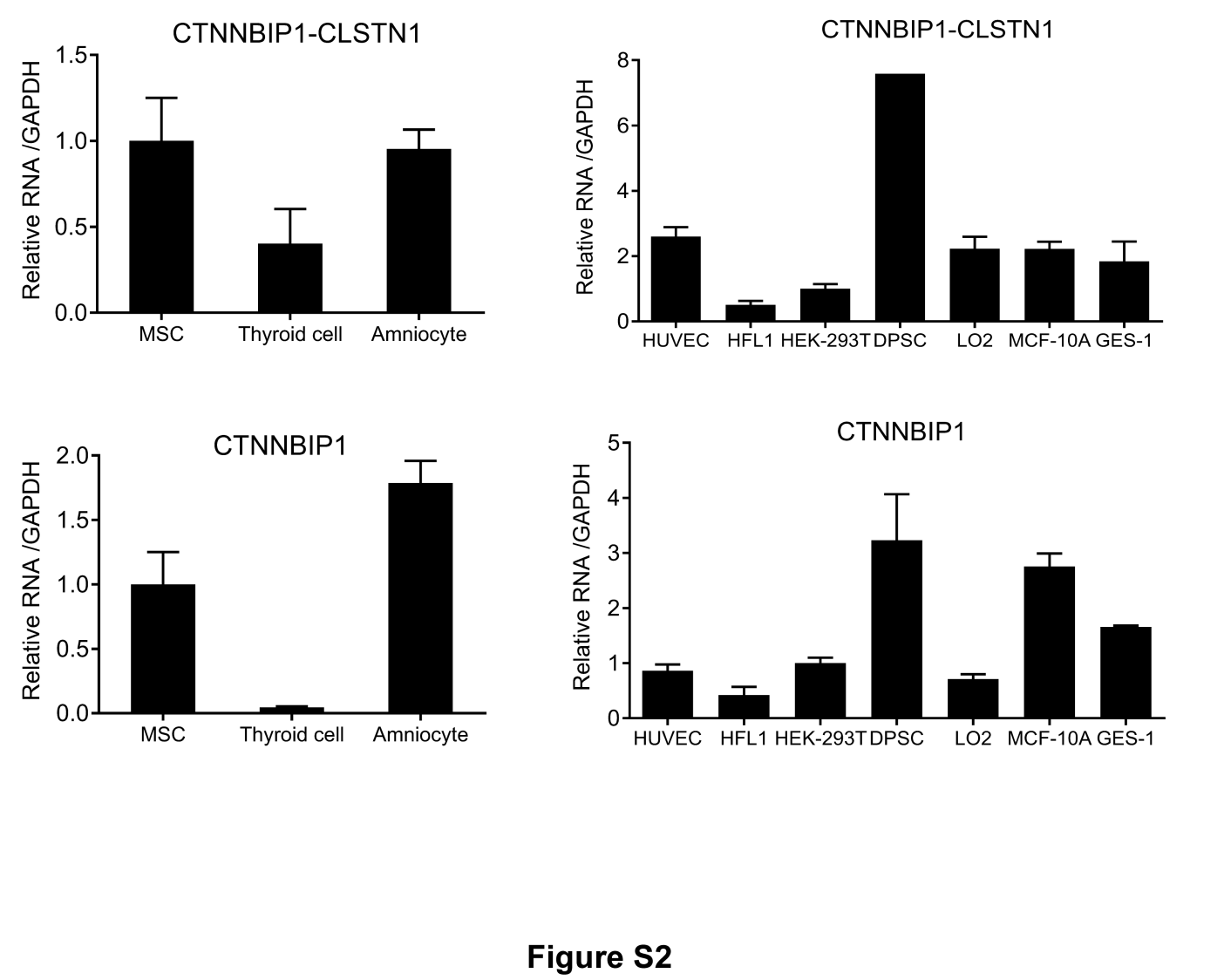


**Supplementary Figure S2. Detection of *CTNNBIP1-CLSTN1* and wild-type *CTNNBIP1* in various human cell lines.** The relative expression levels of *CTNNBIP1-CLSTN1* and *CTNNBIP1* in 10 noncancer human cell lines were measured by qRT‒PCR, normalized to that of the internal control *GAPDH* and further normalized to that in MSCs (left) or HEK-293T cells (right).

**
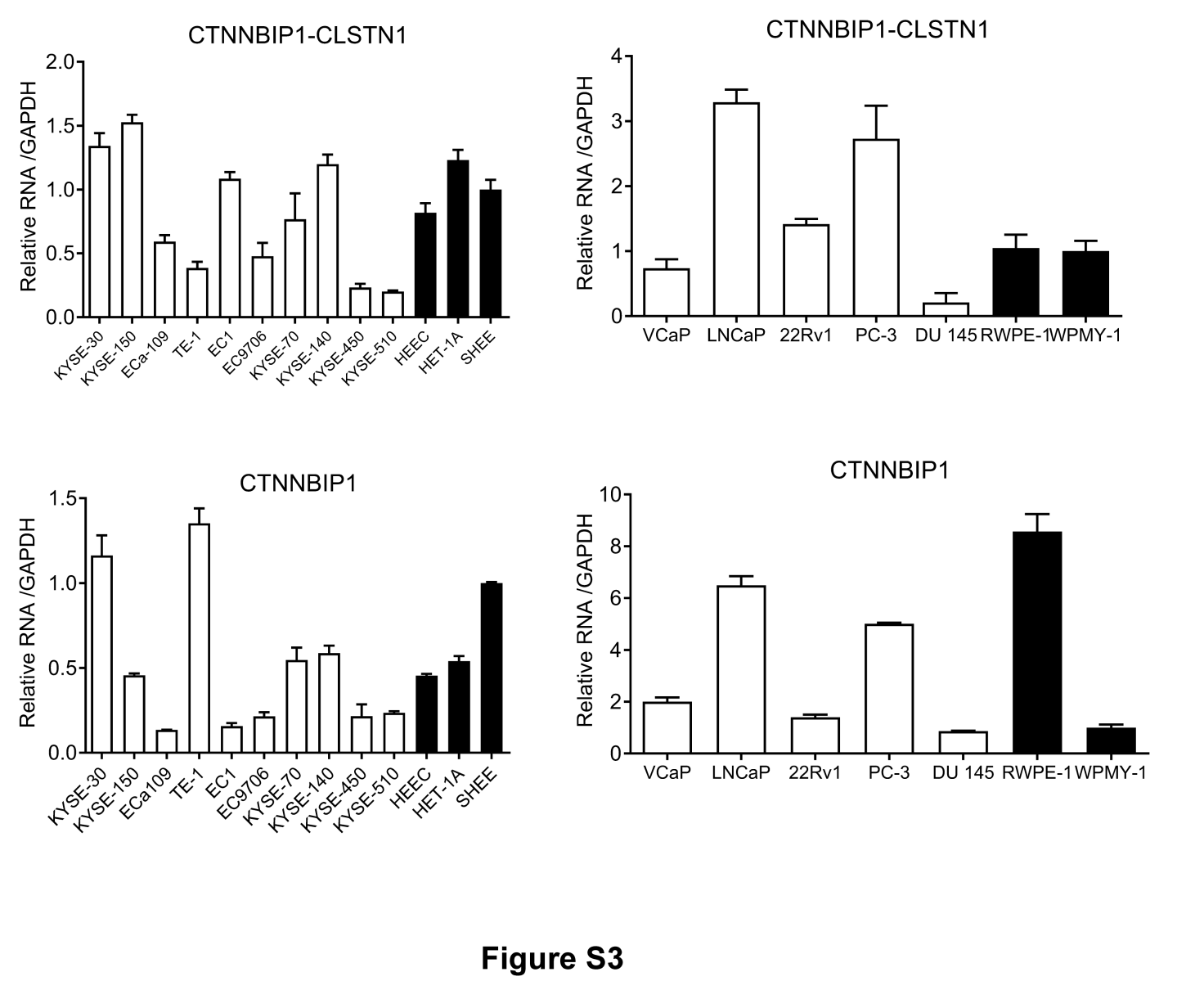
**

**Supplementary Figure S3. Detection of *CTNNBIP1-CLSTN1* and wild-type *CTNNBIP1* in various esophageal and prostate cell lines.** Relative expression levels of *CTNNBIP1-CLSTN1* and *CTNNBIP1* in five human noncancer cell lines and 15 cancer cell lines. The black bars indicate human noncancer cell lines, and the white bars indicate human cancer cell lines. The expression of the transcripts was first normalized to that of the internal control, *GAPDH*, and then further normalized to that in SHEE (left) or WPMY-1 (right) cells.

**
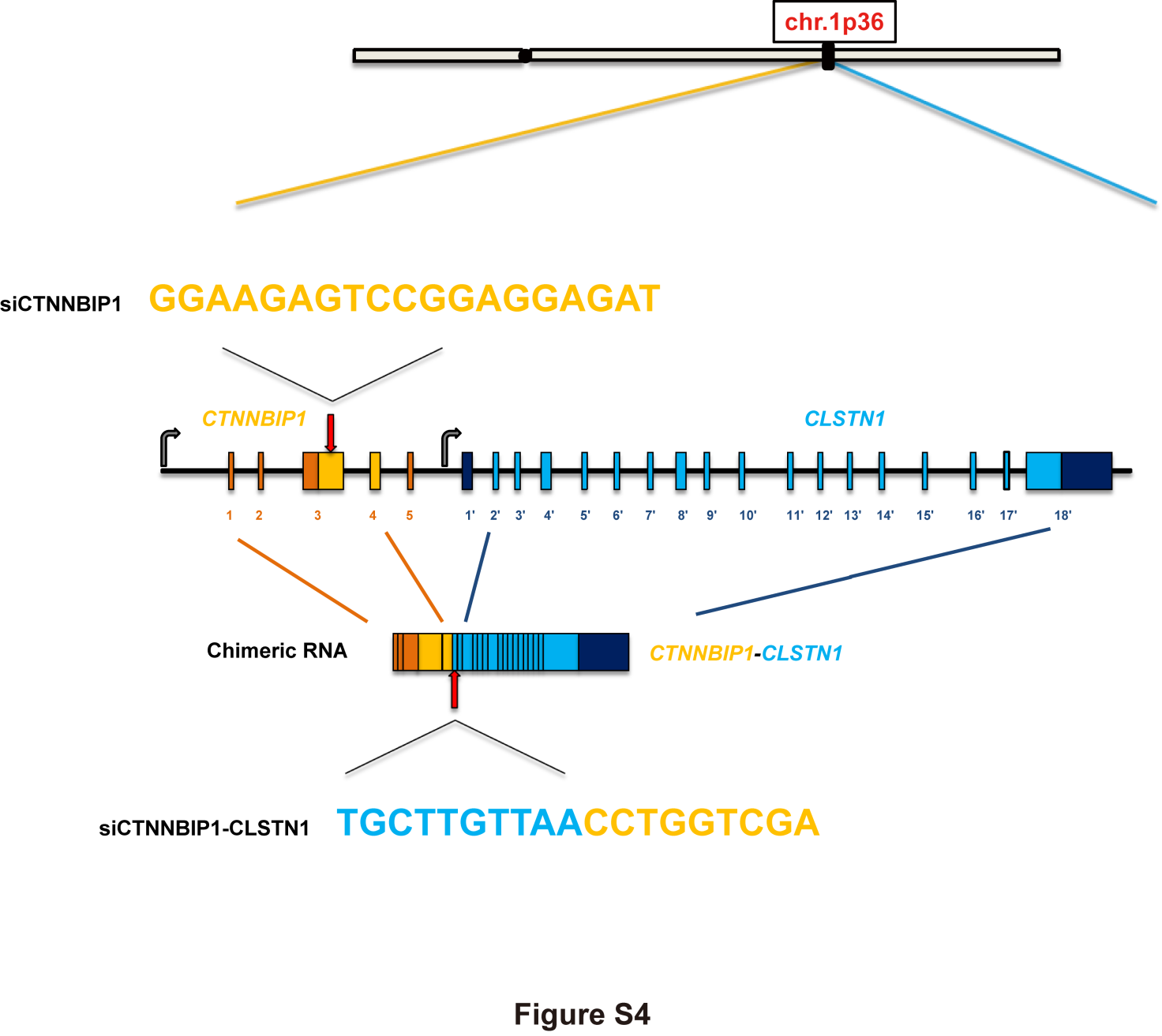
**

**Supplementary Figure S4. Target sites and sequences of siCTNNBIP1-CLSTN1 and siCTNNBIP1.** The blocks indicate exons, and the lines indicate introns and the intergenic region. Orange represents the exons of *CTNNBIP1*, and blue represents exons of *CLSTN1*. As shown in the figure, siCTNNBIP1 targets the third exon of *CTNNBIP1*, whereas siCTNNBIP1-CLSTN1 targets the junction site including the fourth exon of *CTNNBIP1* and the second exon of *CLSTN1*. Lighter colors indicate the coding regions included in the *CTNNBIP1-CLSTN1* expression construct.


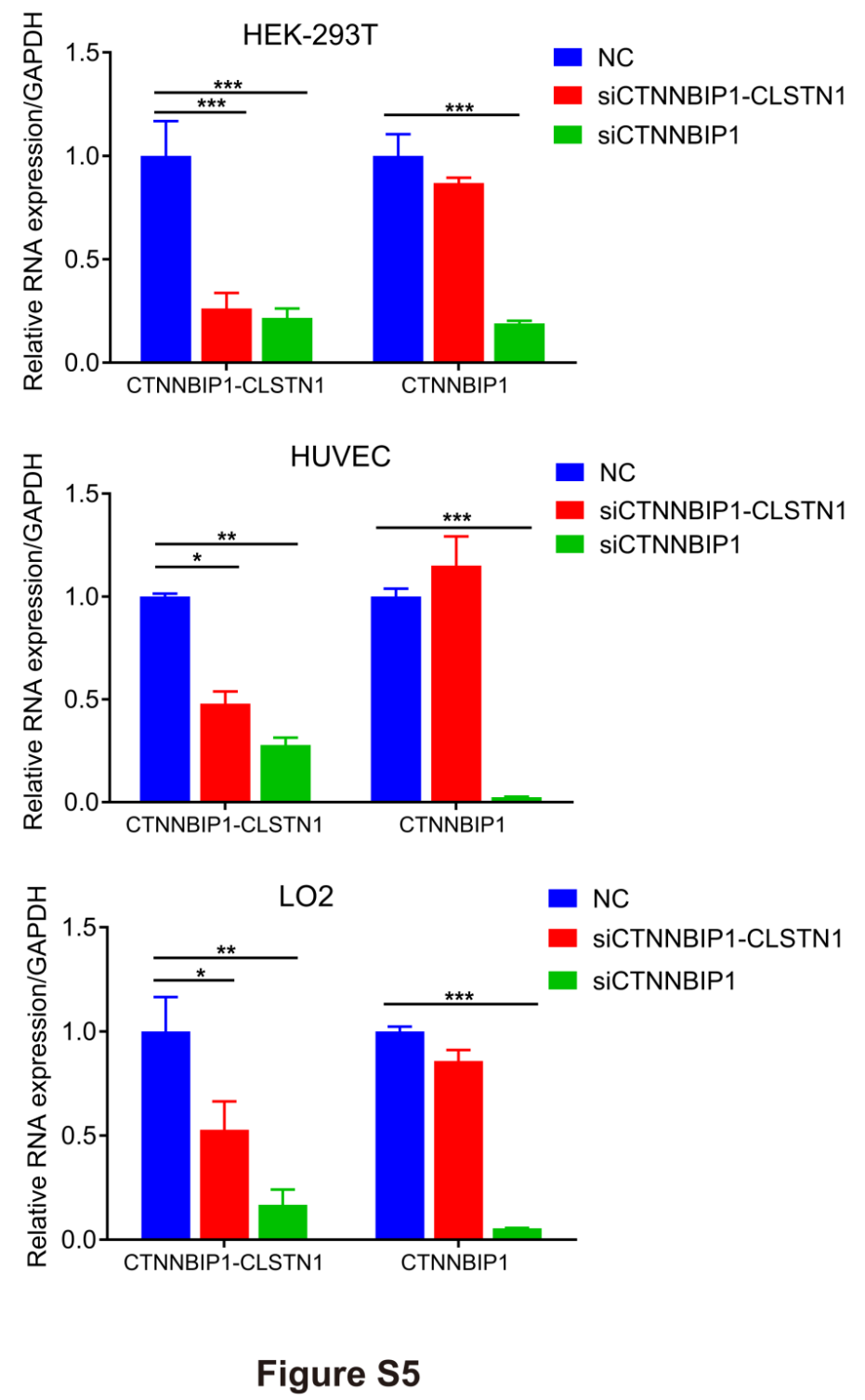


**Supplementary Figure S5. Effects of siCTNNBIP1-CLSTN1 and siCTNNBIP1.** 293T cells, HUVECs and LO2 cells were transfected with siRNA targeting the fusion RNA (siCTNNBIP1-CLSTN1), siRNA targeting wild-type *CTNNBIP1* (siCTNNBIP1) and negative control siRNA (NC). qRT‒PCR was used to measure the expression levels of *CTNNBIP1-CLSTN1* and *CTNNBIP1* 48 hours after siRNA transfection. They both significantly knocked down the expression of the fusion RNA, and siCTNNBIP1 caused an obvious reduction in the expression of wild-type *CTNNBIP1*. The expression levels of the fusion and wild-type CTNNBIP1 were first normalized to those of the internal control, *GAPDH*, and then further normalized to those in the NC group.

**
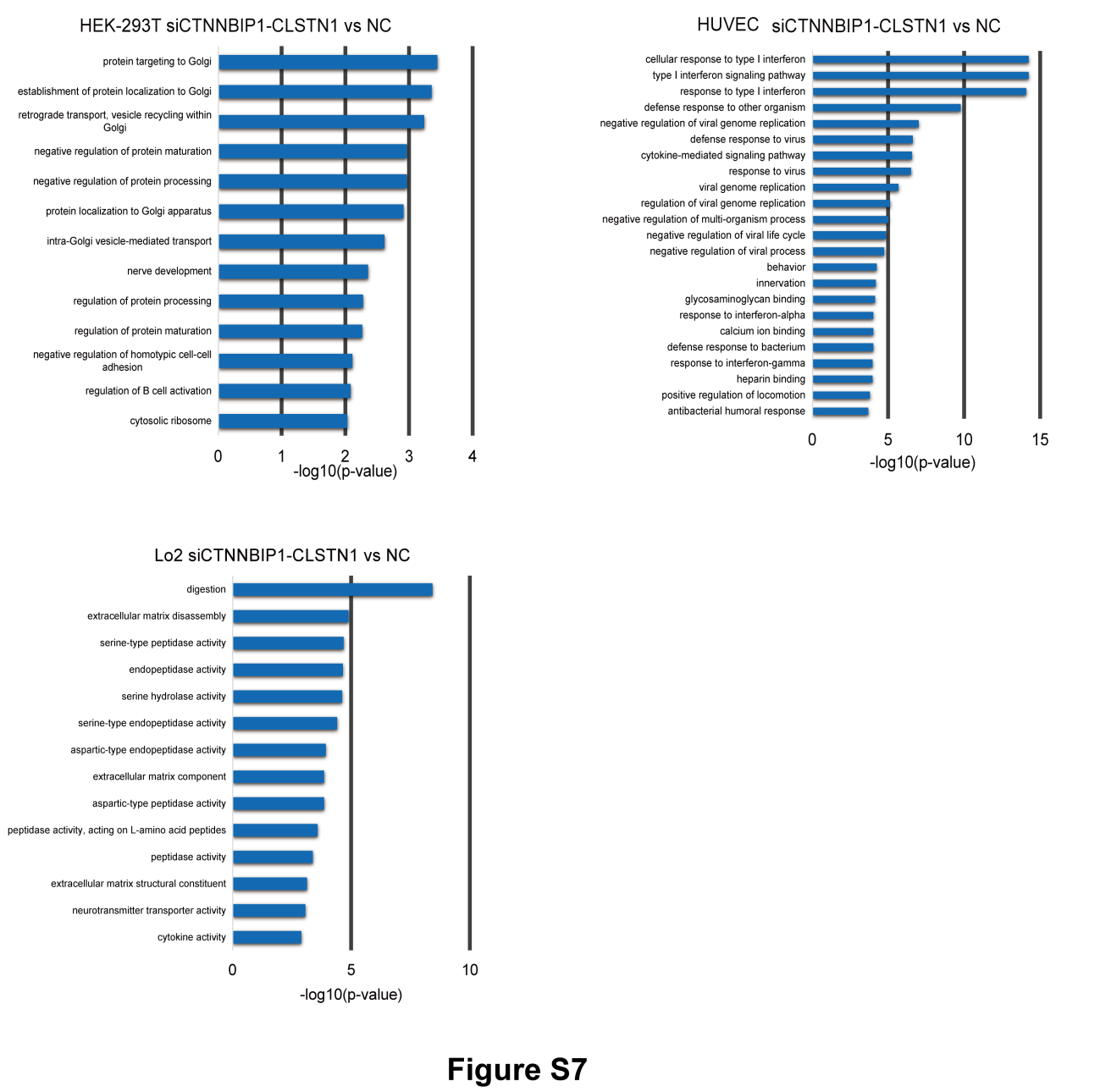
**

**Supplementary Figure S6. Rescue experiments.** HEK-293T cells were first transfected with plasmids expressing *CTNNBIP1-CLSTN1*, plasmids expressing *CTNNBIP1* or the empty control plasmid (Emp.). Twenty-four hours later, the cells were transfected with siCTNNBIP1-CLSTN1, siCTNNBIP1, or the negative control (NC) siRNA. Images were captured every six hours for 24 hours after plasmid transfection, and a cell growth curve was generated based on the cell density. The horizontal axis shows the time after plasmid transfection. The asterisks indicate statistical significance: *P < 0.05. **P < 0.01.

**A
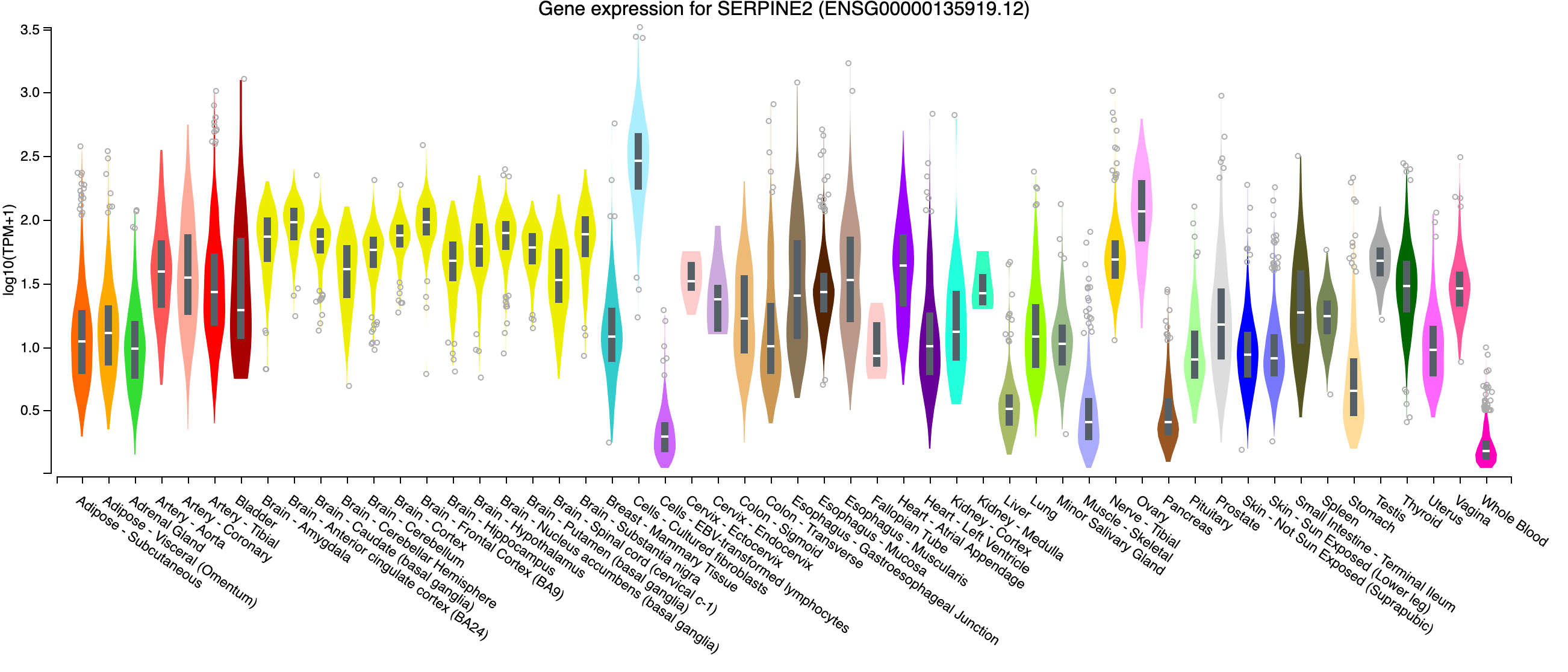
**

**B**

**
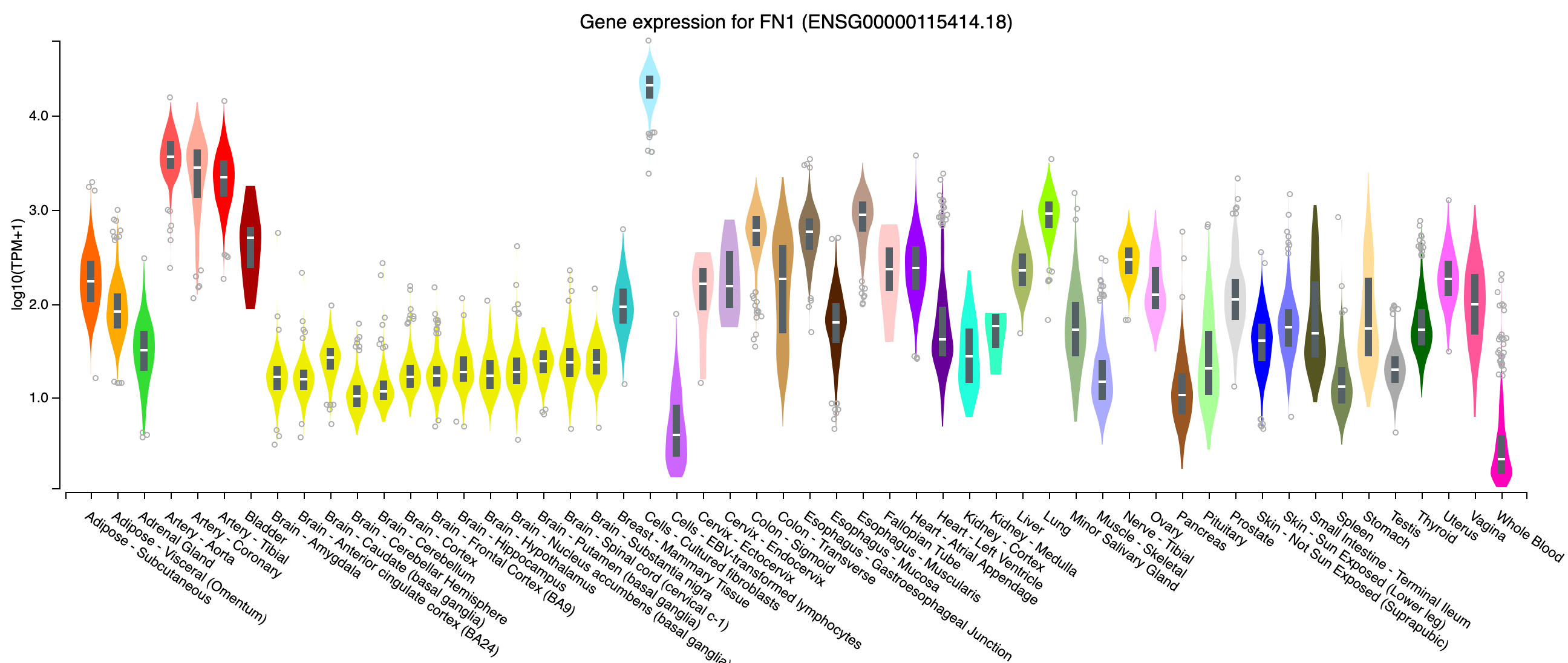
**

**Supplementary Figure S7. Gene Ontology (GO) terms enriched in the differentially expressed genes.** Gene ontology term analyses of differentially expressed genes in the siCTNNBIP1-CLSTN1 group compared with the negative control siRNA group in three cell lines. The statistical significance (-Log10(P value)) of each term is plotted.
